# Teaching evidence based medicine literature searching skills to medical students during the clinical years - a protocol for a randomised controlled trial

**DOI:** 10.1186/1472-6920-11-49

**Published:** 2011-07-28

**Authors:** Dragan Ilic, Katrina Tepper, Marie Misso

**Affiliations:** 1 Department of Epidemiology & Preventive Medicine, School of Public Health & Preventive Medicine, Monash University, Victoria, Australia; 2 Hargrave-Andrew Library, Monash University, Victoria, Australia; 3 The Jean Hailes Foundation for Women's Health Research Unit, School of Public Health & Preventive Medicine, Monash University, Victoria, Australia

## Abstract

**Background:**

Two of the key steps in evidence based medicine (EBM) are being able to construct a clinical question and effectively search the literature to source relevant information. No evidence currently exists that informs whether such skills should be taught to medical students during their pre-clinical years, or delivered to include both the pre-clinical and clinical years of study. This is an important component of curriculum design as the level of clinical maturity of students can affect their perception of the importance and uptake of EBM principles in practice.

**Methods/Design:**

A randomised controlled trial will be conducted to identify the effectiveness of delivering a formal workshop in EBM literature searching skills to third year medical students entering their clinical years of study. The primary outcome of EBM competency in literature searching skills will be evaluated using the Fresno tool.

**Discussion:**

This trial will provide novel information on the effectiveness of delivering a formal education workshop in evidence based medicine literature searching skills during the clinical years of study. The result of this study will also identify the impact of teaching EBM literature searching skills to medical students during the clinical years of study.

## Background

Competency in evidence based medicine (EBM) provides clinicians with the ability to identify, evaluate and integrate evidence into clinical decision making. Two of the five critical steps in achieving competency in EBM are to (i) construct an answerable question from the clinical environment, and (ii) effectively and efficiently search the medical literature to identify the best available evidence to answer the question [1].

Most studies performed to date have focused on evaluating participants' critical appraisal skills in EBM. A systematic review of postgraduate teaching in EBM conducted in 2004 identified improvement in knowledge, critical skills, attitudes and behaviour; however it did not formally evaluate EBM skills in literature searching [2]. A systematic review of EBM teaching from the undergraduate and post-graduate perspective identified one study that reported an increase in formulating clinical questions and searching the literature skills in medical residents attending a formal EBM workshop [3,4]. Similarly, a trial in undergraduate medical students comparing a workshop to didactic EBM teaching reported an increase in skills to construct a clinical question and perform a literature search in students attending the workshop compared to the didactic teaching [4,5].

Various training modules and courses in EBM have now commonly been implemented across medical schools worldwide [6]. The aim of such EBM programs is to provide an integration of knowledge, cognitive skills and behaviour that promotes life-long learning for future medical graduates [4]. Several studies have reported that training undergraduate medical students in EBM literature searching skills using a variety of interventions ranging from individual workshops to ongoing didactic lectures, is associated with an improvement in literature searching skills [5,7-9].

Whilst various studies have identified the positive impact of EBM workshops on students, none have identified whether specialised searching skills are more effectively taught to medical students solely during their pre-clinical or clinical years; or a combination of both. The level of clinical maturity of students can affect their perception of the importance and uptake of EBM principles in practice. A study of junior doctors' knowledge and beliefs in EBM identified a belief that EBM was an essential skill relevant to their clinical practice, despite few having partaken in formal training in the principles of EBM [10]. Conversely, first year medical students who have not been exposed to the clinical environment have reported to perceive EBM as a static discipline, which is not as relevant to clinical medicine [11].

It is important to identify when certain aspects of EBM, be it constructing a question, searching the literature or critically appraising evidence, should be delivered within the undergraduate program in order to ensure that students obtain a satisfactory level of competence in EBM and are able to implement it throughout their clinical years.

## Methods/Design

### Aim

The aim of this randomised controlled trial (RCT) is to determine the effectiveness of delivering a formal education workshop in evidence based medicine literature searching skills to undergraduate medical students during the clinical years of study. The specific objectives of the study are;

1. Compare the competency in EBM literature searching skills of medical students who participate in a formal workshop at the beginning of their clinical year, to medical students who have participated in introductory EBM literature search skills sessions in the preclinical years only; and

2. Determine students self perceived competency in EBM literature searching skills.

### Design

This study is a randomised controlled trial conducted at Monash University, Australia.

### Settings and participants

The study will be performed at the medical library at the Clayton campus of Monash University. Participants for the study will be third year medical students undertaking the Monash University MBBS degree at the Monash Medical Centre, Dandenong or Casey hospitals. Each of these hospitals are teaching hospitals, which form the 'Southern Health' network of hospitals.

The Monash University MBBS degree is a five year undergraduate course. Students within this course spend the first two years based at the University outside of the clinical environment. In the first two years of the MBBS course the students undertake introductory sessions in the principles of evidence based medicine literature searching. At the commencement of the third year students are solely based in the clinical environment, spending their entire teaching year based at one teaching hospital. From years three to five in the course students are only placed within a clinical learning environment.

The Monash MBBS undergraduate degree, and by extension the students, are comparable to similar MBBS degrees within Australia, and internationally, that train undergraduate students in the principles of EBM across preclinical and clinical years of the degree [12].

### Recruitment

Third year medical students will be randomised to either a formal workshop in EBM literature searching skills as part of the EBM unit within the MBBS course or to a control group. Participants must be a third year Monash MBBS student to be eligible for the trial. Students who are unwilling to participate in the study will be excluded from the recruitment process.

### Randomisation

**Figure 1:**
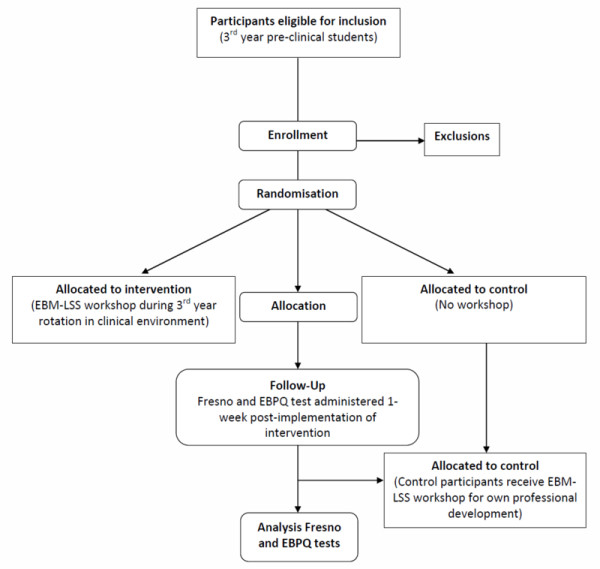
**Flow diagram of proposed randomised controlled trial**.

Participants will be randomly assigned independently by the 'Southern Health' clinical site administrator by block randomisation to either the intervention or comparison groups. A computer random number generator will be used to generate a randomisation list in blocks of four (Figure 1).

### Intervention

Students randomised to the EBM literature searching skills (EBM-LSS) workshop will receive a two hour workshop within the computer laboratory at the Hargrave-Andrew Library at the Clayton campus of Monash University. The EBM-LSS workshop will consist of a 30-minute formal presentation by the subject librarian, an interactive computer based searching session, and self-directed learning tasks (with the subject librarian to provide support when requested). Students randomised to the control group will not receive the workshop. Students in the control group will attend the EBM-LSS workshop once assessment of all outcomes across the intervention and control groups has been completed.

### Outcome measures

The primary outcome to be measured in this study will be competency in EBM literature searching skills. This will be measured by using the previously validated Fresno tool [13]. Additionally, student self perceived competency in EBM literature searching skills will be assessed using the previously validated Clinical Effectiveness and Evidence Based Practice Questionnaire (EBPQ) [14]. These outcomes will be measured across both intervention and control groups 1-week post-implementation of the intervention.

### Blinding

Blinding of investigators and participants is not possible as the subject librarian and students will be aware of the allocated arm. The outcome assessor and data analyst will be kept blinded to the allocation.

### Analyses

#### Sample size calculation

We referred to a previous study, which implemented the Fresno test to assess searching skills, to estimate our sample size [15]. This previous study identified that a mean difference of 13 points on the Fresno test to be statistically significant in identifying competency between groups in effective EBM literature searching. A mean difference of 13 points, with a standard deviation of 10, is meaningful to discriminate between 'novice' and 'expert' users of EBM principles [13]. We determined that with a power of 90%, alpha of 0.05, the required sample size per each group is 21, for a total sample size of 42 participants.

Multiple regression analysis will be used to determine the ability of certain variables (such as hospital site, previous exposure to EBM, perceived research ability and country of origin) to predict competency in EBM literature searching. We determine that with a power of 80%, alpha set at 0.05, and a medium effect size (f^2 ^= 0.15), a sample size of 84 is required to detect a significant model. Therefore, accounting for a potential 10% drop-out rate, a minimum of 100 participants, 50 per each group, will be recruited for the study.

#### Analyses

Data will be analysed using the principle of intention-to-treat. Mean difference in EBM literature searching skills competency between the intervention and control groups, as determined by the Fresno tool, will be explored using a Student's t-test. Additional analyses will include descriptive statistics to characterise participants in terms of self perceived competency in EBM literature searching skills. Linear and logistic regression will be used to analyse continuous and dichotomous data respectively.

### Ethical approval

Ethical approval for this study was granted by the Monash University Human Research Ethics Committee in November 2010.

## Discussion

The primary purpose of this RCT is to identify the effectiveness of delivering a formal workshop in EBM literature searching skills to third year medical students entering their clinical years of study. This study will also assess the impact of clinical maturity of medical students with respect to their knowledge and uptake of EBM literature searching skills. The trial will also provide valuable information with regard to the students' self perceived competency in EBM literature searching skills and what impact this has upon their actual competency.

Previous studies have suggested that clinical maturity, perceived relevance in the clinical environment and continued practice within this context may influence a student's competence in EBM skills [10,11,16,17]. This study has the potential to further explore these issues and provide novel information with regard to student competency in EBM skills, which may assist in the formulation and refinement of existing medical curricula within the EBM context.

## Competing interests

Dragan Ilic is the coordinator for the EBM unit within the Monash University MBBS program. The authors declare no other competing interests.

## Authors' contributions

All authors have had substantial intellectual contribution to this protocol. DI and KT conceived the design of the study. DI wrote the first and final drafts of the protocol. KT and MM contributed to the drafting process of the protocol. All authors have contributed to revising the protocol for intellectual content. All authors have read and approved the final manuscript and given final approval for the manuscript to be published.

## Pre-publication history

The pre-publication history for this paper can be accessed here:

http://www.biomedcentral.com/1472-6920/11/49/prepub
